# Physical activity levels and its associated factors among adults in Vihiga county, Kenya

**DOI:** 10.1371/journal.pgph.0004651

**Published:** 2025-05-28

**Authors:** Miriam Bosire, Doreen Mitaru, Joanna Olale, Schiller Mbuka, Melvine Obuya, Rodgers Ochieng, Boniface Oyugi, Erastus Muniu, Joseph Mutai, Divya Parmar, Lydia Kaduka, Seeromanie Harding

**Affiliations:** 1 Center for Public Health Research, Kenya Medical Research Institute, Nairobi, Kenya; 2 Center for Clinical Research, Kenya Medical Research Institute, Nairobi, Kenya; 3 Faculty of Health Sciences, University of Nairobi, Nairobi, Kenya; 4 School of Population Health Sciences and School of Life Course Sciences, King’s College London, London, United Kingdom; African Population and Health Research Center, KENYA

## Abstract

Sedentary lifestyle is a major risk factor for cardiovascular diseases (CVDs) which account for 8% of Kenya’s non-communicable disease (NCD) burden. Prevalence of physical inactivity remains high globally. There is paucity of data on physical activity levels in rural Sub-Saharan Africa to inform effective interventions. This study sought to establish levels and factors associated with physical activity in a rural population in Kenya. This was a cross-sectional study in Vihiga, a predominantly rural County in Kenya. Participants were adults aged ≥18 years drawn from four community markets. Stratified sampling by ecological zones and rural/urban status was used to select the four markets and Sampling the Next Customer Exiting the Market method for the respondents. Researcher administered e-questionnaire adapted from International Physical Activity Questionnaire (IPAQ) was used to collect data. Physical activity was calculated as the sum of all Metabolic Equivalents (MET)-minutes/week. Multivariable binary logistic regression analysis was used to identify correlates of physical activity. Out of the total 375 (m: 49%; f: 51%) participants, 27% were physically inactive (m: 22%; f: 32%;) and 42% engaged in low level physical activity. Majority of the respondents (75.5%) engaged in transportation-related physical activity while 32% engaged in leisure physical activities. The odds of being physically inactive were 1.93 times higher for females, 2.62 higher for those aged ≥65 years, and 3.62 higher for those with high health literacy. 48% with high health literacy were in the early working age group (15–24 years). Majority (53%) received health information from healthcare workers, especially for the 60% physically inactive participants. This study highlights the need for targeted community interventions to address the observed physical inactivity especially among women and older adults in rural Kenya.

## Background

Sedentary lifestyle is one of the major risk factors for non-communicable diseases (NCDs) such as cardiovascular diseases (CVDs), a major contributor to global morbidity and mortality. Despite ample evidence linking physical inactivity with chronic disease, a significant proportion of the global population remains physically inactive [1], fuelled by increasing rates of industrialization and enhanced technologies. The global prevalence of physical inactivity is estimated at 27.5% [2], and accounts for approximately 3 million deaths and 6–10% of chronic diseases such as coronary heart disease and diabetes [3]. At least one in every four adults globally does not meet the recommended levels of physical activity (PA) despite it being one of the leading modifiable risk factors for NCDs [4]. Physical inactivity in developed countries is reported at 36.8%, with women being less active than men [4]. In sub-Saharan Africa (SSA), physical inactivity is estimated at 22% [5] with some regions reporting levels as high as 46% [6]. In Kenya, findings from the 2015 national STEPWise survey reported 11% low levels of PA and 7.7% physical inactivity [7,8], while a 2022 WHO report shows the prevalence of physical inactivity to be 14% in male and 17% in female adults between 18 and 70 years old [9].

The CVD burden varies across regions in Kenya with previous studies reporting higher prevalence of diabetes and hypertension in urban dwellers than rural populations [10–12]. However, recent studies show an increasing prevalence of hypertension and diabetes in rural settings [13] attributed to nutrition transition, accompanying urbanization, and inaccessibility to CVD preventive healthcare services. Physical activity, defined as any bodily movement produced by a person’s skeletal muscles that uses energy, includes activities undertaken while playing, working, carrying out household chores, engaging in recreational pursuits, and traveling [14]. Effective physical activity yields remarkable outcomes in prevention and management of CVDs [15]. Evidence shows physical activity reduces CVD risk by 30 – 50% [16,17]. At least 20–30 minutes/day of moderate to vigorous physical activity is recommended for adults, with slight variations for children and older adults, for at least five days per week [16,18].

Countries are encouraged to take pro-active measures in developing culturally and socially acceptable policies that promote physical activity informed by local data. There is paucity of county-specific data on physical activity in Kenya, especially from rural areas, to inform planning at county and national level. This study sought to assess levels and factors influencing physical activity among adults in a rural set-up in Kenya. Findings are expected to provide insights on context specific factors associated with physical activity among rural populations in Kenya to inform culturally sensitive interventions and future research.

## Methods

### Ethics statement

The study was implemented following ethical approval from KEMRI Scientific and Ethics and Review Unit (KEMRI/SERU/CPHR/003/3862) and study permit from the National Commission for Science, Technology and Innovation (NACOSTI) (NACOSTI/P/19/376). Permission to conduct the study was sought from the Vihiga County Commissioner and County Director of Health. Written informed consent was also sought from the participants prior to their participation.

### Study design

This was a cross-sectional study in four community markets in Vihiga County, Kenya.

### Study area

The study was done in two rural markets (Mudete and Mwichio) and two urban markets (Chavakali and Esibuye) in Vihiga County, Kenya. Vihiga is a predominantly (98%) rural county situated in the western part of Kenya. It has a population of approximately 600,000 (male: 48%; female: 52%) with population density of 1,047 persons/Km^2^ [19]. The county poverty index is 38.6% compared to the national average of 36.1%, county adult literacy level is at 93.8% compared to national levels at 78%. The major economic activities are cottage industries, small scale subsistence farming, tea farming, wholesale and retail trade, quarrying and mining [20]. Control and prevention of NCDs are among the top county priority programs for health for the next five years [20].

Markets were chosen as they serve as the main social hubs for the community attracting different community sectors. Vihiga County has 19 major markets with typical market days. Selection of the four markets was based on agro-ecological zones, rural/urban status, and market size. Vihiga County has upper and lower agro-ecological zones characterized by differences in rainfall and soil patterns, which contributes to variation in farming activities and food availability. Although the county is predominantly rural, consideration was made for potential differences between rural and relatively urban areas.

### Study population

The study targeted adults found in selected markets during data collection days and times.

#### Inclusion criteria:.

The study included adults aged 18 years and above who had been residents in the county for at least 2 years and consented to be part of the study.

#### Exclusion criteria:.

The study excluded those who were below 18 years of age, had not been residents in the county for at least two years and those who did not consent to participate in the study.

### Sample size calculation

The study used the sample calculated for the parent HEKIMA feasibility study [21,22] designed to assess effectiveness of market-based health kiosks in improving health literacy, behaviour change and cardiovascular outcomes. Level of health literacy was unknown. Data from rural Zambia [23] was used to assume a health literacy level of 15–20%, and intra-class correlation coefficient (ICC) between markets of 0.011. To detect an increase in health literacy of 20%, with 80% power and a 5% significance level, after inflating for the cluster design effect {[1+(m − 1)ρ] where m is sample size per cluster and ρ is the ICC}, and inflating for 25% estimated attrition, a minimum sample of 128–143 was derived. For ease of allocation, this was rounded off to 160 participants per market (minimum of 80 participants per market).

The following Casagrade et al formula [24] with adjustment for design effect (due to cluster sampling) was used for sample size calculation:-


$$\mathrm{\backslash [}n\;=\frac{{\left\{Z_{1-\alpha /2}\sqrt{}2p(1-p)+Z_{1-\beta }\sqrt{}p_{1}(1-\;p_{1})+p_{2}(1-p_{2})\right\}}^{2}\;}{{\left(p_{1}-p_{2}\right)}^{2}}[1+(m-1)\rho ]\mathrm{\backslash ]}$$

Where:

- p is the average of p_1_ and p_2_
- Z_1-α/2_ is standard errors from the mean corresponding to 95% confidence interval
- Z_1-β_ is power of the test
- P_1_ is proportion in health literacy in comparison group
- P_2_ is proportion in health literacy in intervention group
- m is sample size per cluster (assumed equal across all clusters)
- ρ is the intra-cluster correlation coefficient (ICC) which measures the correlation between observations within the same cluster.

### Sampling procedure

#### Markets sampling.

Stratified sampling (stratified by ecological zones and rural/urban status) was used to select the four markets.

#### Respondent sampling.

The exit interviews (interviews at the point of customer exit from a market) approach was applied using Sampling the Next Customer Exiting the Market method. The method is based on intercepting customers as they leave market places and interviewing them. The interviewer (s) arrive at the market place and start screening those exiting the market for eligibility. The first eligible customer is then interviewed. At the end of interview with the 1^st^ eligible customer, next customer exiting the market is recruited in the same manner as the first and the process continues until the required sample size is achieved.

To minimize selection bias, data was collected on various market days (typically 2 days/week) for 2 weeks and at various times from mid-morning to evening to ensure that both market sellers and users were sampled. This paper reports findings from the baseline market survey done between 12^th^ October 2021 and 23^rd^ October 2021.

### Data collection

Data on physical activity was collected using a researcher administered e-questionnaire adapted from International Physical Activity Questionnaire (IPAQ) and used to collect data on physical activity levels of the participants. Interviews were done in both English and Kiswahili depending on the respondent’s preference. The IPAQ [25] allowed assessment of physical activity levels across different sets of domains including work related (carrying/lifting heavy loads, construction work); transport related (walking, cycling), leisure related (sports, gyming, jogging/running) and domestic related (digging) physical activities.

### Data management

Data collection was done using the Redcap software [26] and stored on a KEMRI cloud server in Comma Separated Variables (CSV) file formats. The data was downloaded in excel format and exported to SPSS Version 22 for data analysis. Data cleaning and validation was done prior to statistical analysis.

### Computation of indices and other variables

#### Wealth index.

The household’s wealth status was determined from key household asset ownership variables, which were analyzed using Principal Component Analysis (PCA) [27]. The generated factor scores (weights) for each of the assets were summed and ranked into tertiles as poor, medium, and rich based on the lower, middle, and higher score tertiles in that order.

#### Health literacy.

Health literacy was measured by the Test of All Aspects of Health Literacy Scale (AAHLS). Ten questions were asked to assess the functional, interactive and critical levels of health literacy [28]. Classification of health literacy followed the Sarah J. Schrauben and Douglas J. Wiebe [23] method where Health Literacy factor scores are re-classified into tertiles. Factor analysis with oblique rotation was used to extract a single factor that represented health literacy (HL). The generated factor scores (continuous variable) were then reclassified into tertiles to represent Low, Medium, and High health literacy.

#### Physical activity categories using continuous scoring or Metabolic equivalent of task (MET minutes per week).

The Metabolic Equivalent of Task (MET), is a unit used to estimate the amount of oxygen and calories used by the body during physical activity. One MET is defined as the amount of oxygen consumed while sitting at rest and is equal to 3.5 ml O_2_ per kg body weight per min. According to IPAQ scoring protocol, MET-minutes/week of specific activity (walking or moderate intensity activity or vigorous intensity activity) is computed as follows:

Multiply MET value of particular activity (3.3 for walking, 4.0 for moderate intensity activity, and 8.0 for vigorous intensity activity) with hours spent in that particular activity (e.g., walking MET-minutes/week at work = 3.3 × walking minutes × walking days at work).

#### Physical activity.

Total physical activity, calculated by the sum of all the MET-minutes/week for all physical activity levels, yielded the total PA MET-minutes/week which was further categorized into physically inactive (<600 MET-minutes/week) and physically active (≥600 MET-minutes/week).

#### Physical activity categories using categorical scoring.

IPAQ was used as a standardized self-report measure of physical activity. Physical activity was categorized into high, moderate and low levels using criteria outlined in IPAQ guidelines for categorical scoring [25].

### Statistical analysis

Data analysis was carried out using SPSS version 22 statistical software. Exploratory data analysis (EDA) was employed at the initial stage of analysis to identify the normal distribution of variables, missing data, and extreme outliers. At bivariate level analysis, demographic/socio-economic variables (Sex, Age, Marital status, Education, Occupation & Wealth index) and physical activity were assessed for association with sex and physical activity categories using Chi-square test. All variables with a P-value < 0.25 in the bivariate analysis were subjected to multivariate analysis to control confounding effects and identify physical activity correlates.

At multivariate level analysis, Binary Logistic regression analysis with logit link function using Backward Likelihood Ratio (LR) elimination method, was performed to determine factors associated with physical activity. The goodness of fit of the model was checked using the Hosmer-Lemeshow test at p > 0.05. Adjusted Odds Ratios (AOR) with 95% Confidence Intervals (CIs), were used to evaluate the strength of statistical association between independent and dependent variables. All tests were two-sided, and variables with P-values <0.05 in the analysis were considered statistically significant.

## Results

### Socio-demographic characteristics

A total of 375 respondents (192 females and 183 males) participated in the study. The age ranged from 18 to 92 years with a median age of 38.8 years (Interquartile range (IQR): 28.4 – 51.8) with majority (65.1%), falling in the age bracket 25–54 years. Slightly over two thirds (68.0%) were married or cohabitating and 60.5% had attained secondary and post-secondary education. Almost half (49.3%) of the participants had a business (self-employed) as their occupation. Regarding differences between sexes, marital status and occupation were significantly associated with sex. Characteristics of the study participants are shown in **Table 1**.

**Table 1:** Socio-demographic characteristics.

Characteristic	Total	Females	Males	
	N = 375	N = 192	N = 183	
	%	%	%	P-value
**Sex**				
Male	48.8	-----	----	----
Female	51.2	----	----	
**Age group in years**				
15-24 (Early working age)	15.5	16.7	14.2	0.743
25-54 (Prime working age)	65.1	64.6	65.6	
55-64 (Mature working age)	12.0	12.5	11.5	
65 plus (Elderly)	7.5	6.3	8.7	
**Marital Status**				
Single	21.9	20.3	23.5	0.014
Married or Cohabitating	68.0	65.1	71.0	
Separated/ Divorced/Widowed	10.1	14.6	5.5	
**Level of education**				
None/Primary 1–4 years	5.6	5.7	5.5	0.892
Primary education 5–8 years	33.9	33.9	33.9	
Secondary education, 9–12 years	41.6	40.1	43.2	
College/University > 12 years	18.9	20.3	17.5	
**Occupation (last 1 month)**				
Unemployed/Student/Housewife	17.6	24.0	10.9	<0.001
Formal employment	9.1	5.2	13.1	
Casual worker	14.4	8.3	20.8	
Business (self-employed)	49.3	52.6	45.9	
Farmer	9.6	9.9	9.3	
**Wealth Index – Tertiles**				
Poor	33.3	32.3	34.4	0.88
Medium	33.3	34.4	32.2	
Rich	33.3	33.3	33.3	
**Health Literacy Levels**				
Low	30.7	34.4	26.8	0.189
Medium	36.0	35.9	36.1	
High	33.3	29.7	37.2	

### Physical activity

Based on self-reporting, 31.2% reported no physical activity that lasted for at least 10-min continuously in any of the three main physical activity domains (work-related, transport-related, and leisure-related). Walking or cycling was the most reported domain at 75.5% while 32% reported engaging in vigorous or moderate levels of leisure related physical activity for at least 10 minutes continuously. 63.5% of the participants reported work that involved either vigorous-intensity (40%) or moderate-intensity activity (23.5%). There was a significant association between sex and vigorous-intensity work related physical activity (p value = 0.004) and between sex and vigorous leisure-related physical activity (p value = 0.003) where more males were engaged in vigorous-intensity work and leisure related activities (47.5% and 32.8% respectively) than females (32.8% and 19.8% respectively) **Table 2**.

**Table 2:** Self-reported physical activity domains.

Physical activity domain	Total	Females	Males	P-value
	N = 375	N = 192	N = 183	
	%Yes	%Yes	%Yes	
Engage in physical activity?	68.8	65.1	72.7	0.114
Work involves vigorous-intensity activity	40.0	32.8	47.5	0.004
Work involves moderate-intensity activity	23.5	24.0	23.0	0.818
Walk or use a bicycle (pedal cycle) for at least 10 minutes continuously	75.5	77.6	73.2	0.324
Do any vigorous-intensity sports, fitness or recreational (leisure) activities	25.9	19.3	32.8	0.003
Do any moderate-intensity sports, fitness or recreational (leisure) activities for at least 10 minutes continuously	6.1	6.3	6.0	0.923

Using the WHO global recommendation on physical activity for health to categorize physical activity, the proportion of physically inactive (<600 MET-min/week) participants was 27.2% with a higher proportion of females (31.8%) compared to males (22.4%). Using the IPAQ [25] scoring system, those with low levels of physical activity were 41.9% (females 35.9%, males 48.1%) **Table 3**.

**Table 3:** Physical activity categories.

Physical activity	Total	Females	Males	P-value
	N = 375	N = 192	N = 183	
	%Yes	%Yes	%Yes	
**Physical activity categories using continuous scoring (MET)**				
Inactive	27.2	31.8	22.4	0.042
Active	72.8	68.2	77.6	
**Physical activity categories using categorical scoring**				
Low	41.9	35.9	48.1	0.048
Moderate	25.9	29.7	21.9	
High	32.3	34.4	30.1	

### Factors associated with low physical activity status

Total MET minutes/week was used to classify participants into physically active (72.8%) and physically inactive (27.2%). Sex, Age, Education, Occupation and Health Literacy Level met the criteria of inclusion into multivariate analysis (p < 0.25) and were subjected to multivariable binary logistic regression analysis to identify physical activity correlates. Physical activity was found to be independently associated with sex, age, and health literacy. The odds of being physically inactive was 1.93 times higher for females compared to males. Those aged ≥65 years had 2.62 higher odds of being physically inactive in comparison to the 15–24 years age group though marginally significant. For health literacy, those with high health literacy had 3.62 higher odds of being physically inactive compared to low health literacy group **Table 4**.

**Table 4:** Factors associated with the level of physical activity among study participants.

	Physical Activity			Parameter Estimates			
Characteristic	Inactive	Active	P-value	AOR^#^	95% CI*		P-value
	N = 102	N = 273					
	%	%			Lower	Upper	
**Sex**							
Men**	40.2	52.0	0.042	1			
Women	59.8	48.0		1.932	1.183	3.153	0.008
**Age group in years**							
15-24 (Early working age) **	17.6	14.7	0.11	1			
25-54 (Prime working age)	62.7	65.9		1.009	0.521	1.952	0.979
55-64 (Mature working age)	7.8	13.6		0.572	0.214	1.524	0.264
65 plus (Elderly)	11.8	5.9		2.617	0.968	7.074	0.058
**Marital Status**							
Single	18.6	23.1	0.292				
Married or Cohabitating	67.6	68.1					
Separated/ Divorced/Widowed	13.7	8.8					
**Education**							
None/Primary 1–4 years**	8.8	4.4	0.034	1			
Primary education 5–8 years	42.2	30.8		0.716	0.240	2.139	0.550
Secondary education, 9–12 years	32.4	45.1		0.397	0.126	1.244	0.113
College/University > 12 years	16.7	19.8		0.384	0.111	1.330	0.131
**Occupation (last 1 month)**							
Unemployed/Student/Housewife**	22.5	15.8	0.097	1			
Formal employment	6.9	9.9		0.811	0.274	2.399	0.705
Casual worker	19.6	12.5		1.247	0.513	3.027	0.626
Business (self-employed)	45.1	50.9		0.817	0.401	1.665	0.578
Farmer	5.9	11.0		0.408	0.134	1.244	0.115
**Health Literacy Level**							
Low**	21.6	34.1	<0.001	1			
Medium	27.5	39.2		1.235	0.651	2.343	0.519
High	51.0	26.7		3.621	1.952	6.718	<0.001
**Wealth index in tertiles**							
Poor	34.3	33.0	0.591				
Medium	36.3	32.2					
Rich	29.4	34.8					

^**#**^ AOR – Adjusted Odds Ratio; *95%CI – 95% Confidence Interval; **- Reference category

### Distribution of health literacy vs. socio-demographic details

Majority of the people with high health literacy were in the early working age group (15–24 years) while the elderly had the least number of people with a high health literacy. There was an association between health literacy and marital status, education, and occupation (p value (< 0.05). There were more males than females with high health literacy (Table 5).

**Table 5:** Distribution of Health Literacy vs Socio-demographic.

	Health Literacy Levels			
Characteristic	Low	Middle	High	P-value
**Sex**				
Men	26.8	36.1	37.2	0.189
Women	34.4	35.9	29.7	
**Age group in years**				
15-24 (Early working age)	17.2	34.5	48.3	0.087
25-54 (Prime working age)	33.2	35.7	31.1	
55-64 (Mature working age)	26.7	42.2	31.1	
65 plus (Elderly)	42.9	32.1	25.0	
**Marital Status**				
Single	14.6	41.5	43.9	0.003
Married or Cohabitating	35.7	35.7	28.6	
Separated/ Divorced/Widowed	31.6	26.3	42.1	
**Education**				
None/ Primary 1–4 years	38.1	23.8	38.1	0.004
Primary education 5–8 years	36.2	25.2	38.6	
Secondary education, 9–12 years	32.1	39.7	28.2	
College/University > 12 years	15.5	50.7	33.8	
**Occupation (last 1 month)**				
Unemployed/Student/Housewife**	18.2	37.9	43.9	0.025
Formal employment	23.5	44.1	32.4	
Casual worker	22.2	31.5	46.3	
Business (self-employed)	38.4	34.6	27.0	
Farmer	33.3	38.9	27.8	
**Wealth index in tertiles**				
Poor	33.6	35.2	31.2	0.572
Medium	31.2	32.0	36.8	
Rich	27.2	40.8	32.0	

Majority of the respondents specifically the unemployed/students/housewives, casual workers and business people received their health information from health care workers (54.5%, 53.7% and 53.0% respectively) **Table 6**.

**Table 6:** Distribution of source of health information for the different occupations.

	Unemployed/Student/Housewife**	Formal employment	Casual worker	Business (self-employed)	Farmer
**Media (Radio/Television)**					
Yes	31.8	29.4	38.9	38.9	36.5
No	68.2	70.6	61.1	61.1	63.5
**Print, Social media & internet**					
Yes	19.7	32.4	13.0	14.6	8.3
No	80.3	67.6	87.0	85.4	91.7
**Health print media (Magazines, Brochures, Pamphlets)**					
Yes	13.6	26.5	14.8	13.0	8.3
No	86.4	73.5	85.2	87.0	91.7
**Healthcare workers**					
Yes	54.5	38.2	53.7	53.0	47.2
No	45.5	61.8	46.3	47.0	52.8
**Friends/Relatives**					
Yes	21.2	2.9	22.2	12.4	5.6
No	78.8	97.1	77.8	87.6	94.4
**Private consultations**					
Yes	10.6	11.8	3.7	8.6	2.8
No	89.4	88.2	96.3	91.4	97.2

Health related information from print, social media and internet had a positive association with physical activity while information from health care workers and friends or relatives had a negative association with physical activity **Table 7**.

**Table 7:** Association between Source of Health Information and Physical Activity.

	Inactive	Active	Chi-Square
**Media (Radio/Television)**			
Yes	34.3	37.4	0.59
No	65.7	62.6	
**Print, Social media & internet**			
Yes	8.8	19.0	0.017
No	91.2	81.0	
**Health print media (Magazines, Brochures, Pamphlets)**			
Yes	11.8	15.0	0.52
No	88.2	85.0	
**Healthcare workers**			
Yes	59.8	48.4	0.048*
No	40.2	51.6	
**Friends/Relatives**			
Yes	31.4	7.3	0.00*
No	68.6	92.7	
**Private consultations**			
Yes	7.8	8.1	0.95
No	92.2	91.9	

*Negative association with Physical Activity

## Discussion

### Levels of physical activity

This study’s results found the prevalence of physical inactivity to be at 27.2% which is much higher than the national prevalence of 7.7% [7] and African prevalence of 22.1% [29]. Increase in sedentary occupations, modern motorized forms of transportation such as use of motorbikes, lack of access to recreational and outdoor spaces for physical activity and cultural norms have been linked to the increasing prevalence of physical inactivity in urban and rural populations in Kenya [7]. In this study, majority of the participants (49.3%) were market traders (self-employed), whose nature of work entails prolonged seating, standing or minimal movement around their market stalls [30], which may expose them to sedentary lifestyle. Popularising transport-related physical activities such as walking and cycling may provide low-cost culturally sound opportunities for designing cardiovascular health interventions in Vihiga and similar settings [8,31–36].

Incorporating physical activities in traditional community practices has been shown to increase physical activity [37]. Cultural and traditional dances for instance have been shown to promote physical activity among the elderly, minority groups and females [38–40]. Vihiga County is known for its rich cultural heritage which includes folk music, traditional dances such as *isikuti/ingoma* (a traditional dance that involves rhythmic footwork and body movement) and games such as football, rugby and volleyball [20], commonly partaken in community, county and national functions, and which serve as a medium of communication, bonding and exchange. There is the opportunity to harness such heritage and cultures to improve awareness, knowledge exchange and promote transport and leisure related physical activity in rural communities in SSA.

### Factors associated with physical activity

#### Physical activity and sex.

Sex was independently associated with physical activity with the odds of females being physically inactive twice that of males. Previous studies have reported similar patterns globally [33,41–44] and in Kenya [7,45,46]. Studies show variations in participation in the different physical activity domains by gender [42]. In this study, there was a notable association between sex and vigorous-intensity work related and vigorous leisure-related physical activity where more males were engaged than females. Similar findings have been found [36,42,44], where vigorous-work and leisure related physical activities were common in males, and moderate intensity physical activities in females. Such trends can be attributed to societal perception of high-intensity physical activities as being unfeminine and weight gain as prestigious [47]; cultural norms and gender roles where women are often expected to manage the home in addition to any other form of employment [48,49]; and negative stereotypes surrounding physical activity, e.g., dress codes often seen as short and revealing, which may discourage women from participating in outdoor activities [50]. Understanding such perceptions are key to tailoring opportunities for safe, accessible, and culturally appropriate physical activities to bridge the gender gap in physical activity and support behaviour change. The high mobile phone penetration in Kenya [51] presents the opportunities in ICT to raise public awareness and education on the importance of physical fitness across gender and lifespan.

#### Physical activity and age.

Increasing age was also associated with physical activity with those aged ≥65 years having higher odds of being physically inactive. These findings agree with other studies reporting less physical activity in older than young participants [52–57] due to reduced muscle mass, strength, flexibility, swiftness and endurance [58]. Other reported barriers include low motivation to exercise, retirement, misconceptions and poor perceptions about physical activity, lack of recreational infrastructure [59,60] and limited physical activity interventions for the elderly [61].

Maintaining physical activity across all stages of life is necessary in ensuring optimal health benefits [62]. Provision of accessible infrastructure and resources for physical activity programmes attractive to the elderly in rural areas is required. A study done in rural Thailand found that having exercise parks and equipment open to the community increased vigorous-intensity physical activity among adults due to the ease of access to these recreational facilities [58]. It is worthwhile considering mapping of market spaces and creating recreational areas and foot paths to encourage physical activity among market users in rural Kenya.

#### Physical activity and health literacy.

In this study, high health literacy was inversely correlated with physical activity, where those with high health literacy level had 3.62 higher odds of being physically inactive compared to the low health literacy group. Our findings contradict most reports on positive or non-existent relationship between high health literacy levels and physical activity [63–65]. The positive association between health literacy and physical activity has been majorly attributed to the understanding that individuals with high health literacy are equipped with skills and abilities to support behaviour change [64]. Evidence suggests that for health literacy to initiate behaviour change such as physical activity, individuals must have high levels of all the three (functional, interactive and critical) health literacy skills, especially the interactive and critical health literacy skills that equips them with knowledge, competencies and motivation to access, understand, assess and apply health information in a way that promotes behaviour change for improved health [63].

In this study, high health literacy was associated with occupation, educational level, and marital status. Casual workers, those in formal employment and the unemployed, students and housewives had higher number of individuals with high health literacy. Similar to other findings, majority reported health care workers as their main source of health-related information [66–68]. However, the study further found that while majority of the participants relied on health care workers as their source for health related information, there was a negative association between health care workers as a source for health related information and physical activity with majority of the physically inactive participants receiving general health information from health care workers. This may be attributed to the women’s likelihood to access health services and to be physically inactive despite receiving health information from health care workers.

It is imperative for the community to be empowered with critical health literacy skills to help them improve physical activity levels. The high number of respondents trusting health care workers as their source of health information shows that health care workers remain a vital communication medium in advocating for CVD prevention in rural settings. Health care workers should be empowered to bridge gaps in health literacy. Collaboration and coordination between health care workers and other health professionals such as fitness experts may help in providing patient-friendly resources and empowering individuals to play an active role in their personal and community health care [69].

### Study limitations

The study was cross-sectional in nature and causal association between physical activity and identified correlates cannot be inferred. The choice of community market as study site has possibility of missing populations that may not frequent the markets thereby affecting adequate population representation. Physical activity measures were self-reported which introduces recall & information bias that may lead to over- or under-reporting of physical activity.

## Conclusion

High levels of physical inactivity were observed in rural Kenya with majority of the population failing to meet WHO physical activity recommendations. Females and older adults exhibited a higher likelihood of physical inactivity. High health literacy was also associated with physical inactivity, potentially reflecting increased awareness of sedentary lifestyle risks without translating to behavioural change. Social factors such as occupation and source of health information influence health literacy and attendant effects on physical activity and cardiovascular health in general.

These findings underscore the need for targeted interventions that consider the influence of social, cultural and economic factors on physical activity. Gendered response cognisant of people’s way of life is required to address physical inactivity in these rural population. Organising regular activities such as dances and sports at community gathering points such as community markets provides an opportunity to promote culturally appropriate interventions accessible to all segments of society. Group activities and workplace wellness initiatives such as short activity breaks, may be introduced to promote incorporation of movement into daily routines and encourage physical activity. Prioritising infrastructural developments such as recreational grounds may help address the observed know-do-gap while culturally sensitive health education programs leveraging trusted sources of information may mitigate the observed barriers to physical activity.

## Supporting information

S1 Checklist Inclusivity on Global Research Questionnaire. (DOCX)
